# Development of a Simple New Method to Detect Oral Malodor Using a Hydrogen Sulfide Detector Tube

**DOI:** 10.1155/ijod/2255278

**Published:** 2025-11-24

**Authors:** Jun Takatori, Nao Suzuki, Takashi Hanioka, Masahiro Yoneda

**Affiliations:** ^1^ Department of General Dentistry, Fukuoka Dental College, Fukuoka, Japan, fdcnet.ac.jp; ^2^ Department of Preventive and Public Health Dentistry, Fukuoka Dental College, Fukuoka, Japan, fdcnet.ac.jp; ^3^ Oral Medicine Research Center, Fukuoka Dental College, Fukuoka, Japan, fdcnet.ac.jp; ^4^ Faculty of Health Care Sciences, Takarazuka University of Medical and Health Care, Takarazuka, Japan, takara-univ.ac.jp

**Keywords:** detector tube, hydrogen sulfide, oral malodor, organoleptic test, volatile sulfur compounds

## Abstract

**Aim:**

Many patients visit dental clinics complaining of oral malodor. However, there is no simple, inexpensive tool for assessing oral malodor. Therefore, this study developed a simple method using a detector tube.

**Materials and Methods:**

A detector tube was created to detect hydrogen sulfide based on the color change of an indicator (GASTEC, Kanagawa, Japan). We confirmed the ability to detect hydrogen sulfide concentrations of 200 ppb, which corresponds to the human olfactory threshold, using standard gas. The hydrogen sulfide detector tube was used to evaluate oral malodor in 42 outpatients aged 16–80 years, and the results were compared with an organoleptic test (OLT) and volatile sulfur compound (VSC) concentrations measured using a portable sulfide monitor.

**Results:**

Comparing the hydrogen sulfide detector tube with the OLT score, the sensitivity was 0.90 and the specificity was 0.74 for OLT score ≧2.75 (*n* = 37). For VSC concentrations measured by the sulfide monitor, the sensitivity was 0.85 and the specificity was 1 at ≧300 ppb (*n* = 41). For OLT score ≧2.75 or VSC ≧300 ppb, which are considered indicators of “clearly noticeable oral malodor,” the detector tube showed a sensitivity of 0.84 and a specificity of 1. The diagnostic performance of the detector tube decreased when evaluating mouth air rather than standard gas, possibly due to the inhibitory effects of humidity and other gases in mouth air. Although it did not correspond to the olfactory threshold, the detector was highly sensitive and specific for determining the level of “clearly noticeable oral malodor”; it was considered a practical, easy‐to‐use tool.

**Conclusion:**

The new hydrogen sulfide detector tube, when used in combination with OLT, should be useful for determining “clearly noticeable oral malodor”.

## 1. Introduction

Oral malodor is the third most common reason for visiting a dentist, after dental caries and periodontal disease [1]. However, it is difficult for patients to accurately recognize their own oral malodor. Whereas some patients become aware of their oral malodor when family members or close friends point it out, many begin to worry about oral malodor based on the gestures and expressions of others. Furthermore, although oral malodor is genuinely present in many cases, some patients experience substantial anxiety even when the odor is not sufficiently severe to be problematic [2, 3]. Therefore, it is important to objectively evaluate the level of oral malodor in patients who complain of it.

There are two methods for evaluating oral malodor: the organoleptic test (OLT) and instrumental tests. Although OLT can be used to examine various odorous components, it is recommended that odor assessments be combined with objective evaluations using instruments; OLT relies on the human sense of smell [4]. Most instrumental tests measure volatile sulfur compound (VSC) concentrations, which are the main components of oral malodor [5]. Simple gas chromatographs include the Oral Chroma (Nissha FIS, Osaka, Japan) [6] and Twin Breasor Ⅱ (iSenLab, Gyeonggi‐do, Korea) [7]; portable sulfide monitors include the Halimeter (Interscan, Chatsworth, CA, USA) [8] and Breathtron (Yoshida, Tokyo, Japan) [9]. Devices that detect reducing gas components (B/B Checker, Taiyo, Osaka, Japan) and ammonia (ATTAIN, Taiyo, Osaka, Japan) are also used [10, 11]. However, most of these devices are expensive, need regular maintenance, and require considerable time to produce measurements, limiting their adoption in dental clinics.

If there were a simple, inexpensive method for determining oral malodor that did not require special maintenance, it could better meet patient needs and assist in deciding when to refer patients to specialist outpatient clinics.

Therefore, in this study, we focused on hydrogen sulfide, the main component of oral malodor, and attempted to develop a device capable of determining oral malodor inexpensively and easily using a hydrogen sulfide detection tube.

## 2. Materials and Methods

### 2.1. Creation of a Hydrogen Sulfide Detector Tube

A simple hydrogen sulfide detector tube was developed using a device that turns red on exposure to 200 ppb of hydrogen sulfide standard gas, the olfactory threshold level for humans [12], at an inhalation rate of 1 L/min for 1 min (total inhaled volume: 1 L) (Figure 1A, GASTEC). To address the reduction in reaction caused by moisture in the mouth, a dehumidifier was placed on the mouth intake side of the detector.

Figure 1(a) The hydrogen sulfur detector tube. (b) Measurement of oral malodor using the hydrogen sulfide detector tube. (c) Positive reaction in the hydrogen sulfide detector tube.

**Figure:**
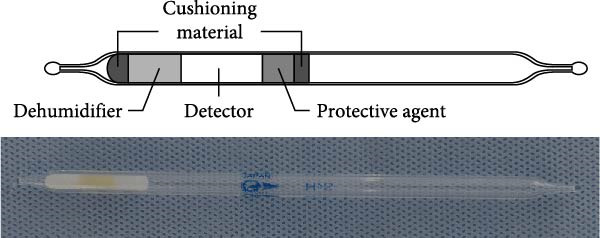
(a)

**Figure:**
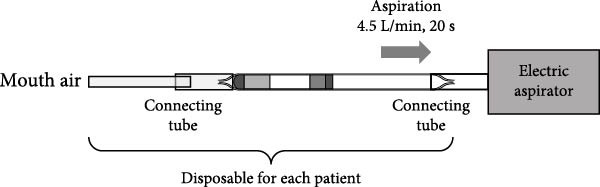
(b)

**Figure:**
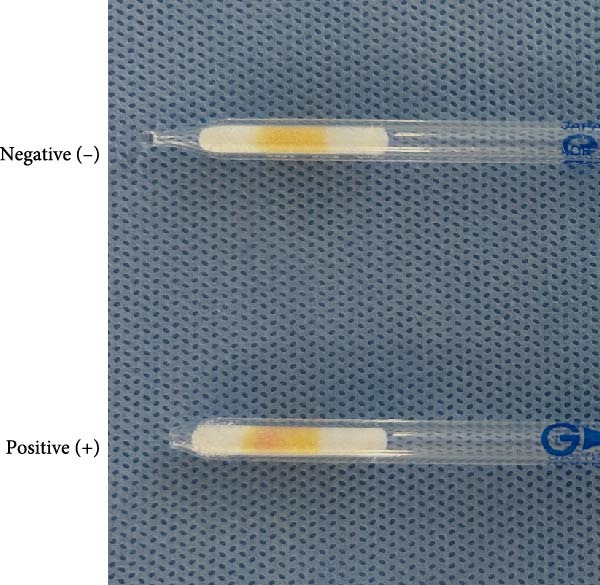
(c)

### 2.2. Clinical Validation

The clinical utility of the detector tube was evaluated in 42 outpatients (aged 16–80 years; 33 women, 9 men) who complained of oral malodor. As standard oral malodor tests, OLT [13] and measurement of total VSC levels using an MS Halimeter (Morita, Tokyo, Japan) were performed. The OLT was scored from 0 to 5 in 0.5 increments. The OLT was conducted twice by one examiner, and the average score was used as the representative value for each individual. For the MS Halimeter, the average concentration (ppb) of three measurements was considered the representative value for each individual. For measurement using the detector tube, mouth air was aspirated at a flow rate of 4.5 L/min for 20s (total inhaled volume: 1.5 L) (Figure 1B). During aspiration, patients were instructed to lightly bite the suction port, gently close their lips, and breathe only through their nose to ensure that mouth air was aspirated and saliva did not enter the suction port. A red color change in the detection agent was judged as positive (Figure 1C). Oral malodor testing was performed at least 2 h after eating or cleaning the mouth.

### 2.3. Ethical Considerations

This study was approved by the Fukuoka Gakuen Ethics Committee (Permission Number 538).

### 2.4. Statistical Analysis

To evaluate the accuracy of the conventional oral malodor test, the Pearson correlation between OLT score and VSC concentration measured with the MS Halimeter was assessed. Sensitivity, specificity, false positive rate, and diagnostic performance were calculated to evaluate the accuracy of the detector tube for OLT score and VSC concentration separately and combined. Based on the definition of an appropriate cutoff value in ROC analysis [14], the distance from the upper left corner of the ROC curve was calculated as the diagnostic performance index, and smaller values were assumed to indicate better performance. Statistical analysis was performed using SPSS Statistics 27 (IBM, Tokyo), with a significance threshold of 5%.

## 3. Results

### 3.1. Levels of Oral Malodor Among Study Participants

Of the 42 participants, one could not be evaluated because her saliva contaminated the detection agent. In addition, four could not undergo the OLT due to infectious diseases or other reasons. Therefore, 37 participants were analyzed using the OLT, and 41 had total VSC levels measured using the MS Halimeter. The correlation between the OLT score and total VSC levels for the 37 participants who underwent both tests, was positive, with a correlation coefficient of 0.80 (*p* < 0.01).

### 3.2. Accuracy of the Hydrogen Sulfide Detector Tube Versus the OLT Score

**Figure 2:**
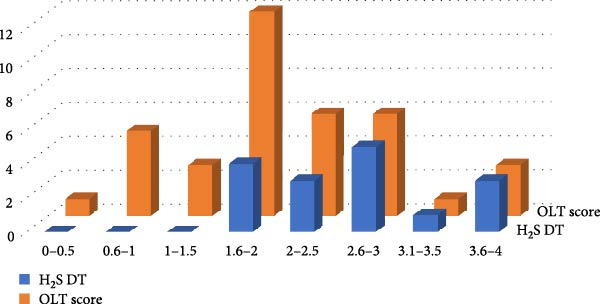
Distribution of OLT scores and corresponding distribution of positive reactions with the hydrogen sulfide detector tube. The X‐axis shows the OLT score (orange bars) and positive H_2_S detection tube (blue bars), and the Y‐axis indicates the number of cases.

Figure 2 shows the distributions of OLT scores and positive results from tests using hydrogen sulfide detector tubes. The most common OLT score was 2, which is the threshold for the presence of oral malodor (12 participants, 32.4%); 10 participants (27.0%) had a score ≧2.75, the threshold for clearly noticeable oral malodor. The hydrogen sulfide detector tube did not show positive reactions below the threshold for oral malodor. However, the positive rate was low for an OLT score of 2 (33.3%) and very high for scores ≧2.75 (90.0%). Table 1 presents the sensitivity, specificity, false positive rate, and diagnostic performance. The hydrogen sulfide detector tube demonstrated excellent diagnostic performance for OLT scores ≧2.75.

**Table 1:** Relationship between judgments using the detector tube and OLT score (*n* = 37)

OLT score	Sensitivity	Specificity	False positive rate	Diagnostic performance ^∗^
≧2.0	0.62	1.00	0.00	0.380
≧2.5	0.75	0.81	0.19	0.314
≧2.75	0.90	0.74	0.26	0.278
≧3.0	0.89	0.71	0.29	0.31
≧3.5	1.00	0.64	0.36	0.36

^∗^Diagnostic performance: lower values indicate better performance.

### 3.3. Accuracy of the Hydrogen Sulfide Detector Tube Versus VSC Levels Measured Using the MS Halimeter

**Figure 3:**
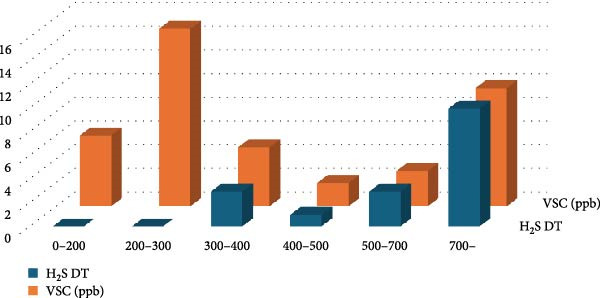
Distribution of VSC concentrations measured using a portable sulfide monitor and corresponding distribution of positive responses with the hydrogen sulfide detector tube. The X‐axis shows the VSC level (ppb) measured by the Halimeter (orange bars) and positive H_2_S detection tube (blue bars), and the Y‐axis indicates the number of cases.

Figure 3 shows the distributions of total VSC levels measured with the MS Halimeter and positive results from the hydrogen sulfide detector tube. The most common VSC level was observed in 15 cases (36.6%) at log 2.3–2.5 (200–300 ppb), but no positive reactions were recorded in these cases using the hydrogen sulfide detector tube. The positive rate was 85.0% for VSC ≧log 2.5 (300 ppb), and all cases showed positive reactions at VSC ≧log 2.7 (500 ppb). Table 2 presents the sensitivity, specificity, false positive rate, and diagnostic performance. The hydrogen sulfide detector tube proved highly effective for assessing VSC ≧log 2.5 (300 ppb) measured by the MS Halimeter.

**Table 2:** Relationship between judgments using the detector tube and VSC concentrations (*n* = 41)

H_2_S (ppb)	Sensitivity	Specificity	False positive rate	Diagnostic performance ^∗^
≧200	0.52	1.00	0.00	0.48
≧250	0.71	1.00	0.00	0.29
≧300	0.85	1.00	0.00	0.15
≧350	0.88	0.92	0.08	0.144
≧400	0.93	0.88	0.12	0.139

^∗^Diagnostic performance: lower values indicate better performance.

### 3.4. Accuracy of the Hydrogen Sulfide Detector Tube Versus Conventional Oral Malodor Judgment Combining OLT and MS Halimeter

Since oral malodor is typically determined using a combination of OLT and instrument analysis, we evaluated the accuracy of the hydrogen sulfide detector tube relative to the combined assessment of OLT and VSC levels measured with the MS Halimeter. Table 3 shows the relationship between oral malodor determinations based on the combination of OLT and MS Halimeter results and those using the hydrogen sulfide detector tube. The hydrogen sulfide detector tube performed well for OLT scores ≧2.75 and/or MS Halimeter values ≧300 ppb.

**Table 3:** Relationship between judgments using the detector tube and the combination of OLT and VSCs (*n* = 37).

Judgment criteria	Sensitivity	False positive rate	Diagnostic performance ^∗^
OLT score ≧2.5 AND/OR ≧250 ppb	0.64	0.00	0.380
OLT score ≧2.75 AND/OR ≧250 ppb	0.67	0.00	0.330
OLT score ≧3.0 AND/OR ≧250 ppb	0.73	0.00	0.270
OLT score ≧2.5 AND/OR ≧300 ppb	0.80	0.00	0.200
OLT score ≧2.75 AND/OR ≧300 ppb	0.84	0.00	0.160
OLT score ≧3.0 AND/OR ≧300 ppb	0.84	0.00	0.160
OLT score ≧2.5 AND/OR ≧350 ppb	0.79	0.06	0.218
OLT score ≧2.75 AND/OR ≧350 ppb	0.83	0.05	0.177
OLT score ≧3.0 AND/OR ≧350 ppb	0.83	0.05	0.177

^∗^Diagnostic performance: lower values indicate better performance.

## 4. Discussion

A new tool was developed in this study for screening oral malodor in private dental clinics, which could be valuable for managing patients’ oral health and referring them to oral malodor specialists. The primary components of oral malodor generated in the oral cavity are volatile sulfur compounds, including hydrogen sulfide, methyl mercaptan, and dimethyl sulfide. Of these, hydrogen sulfide is the predominant odorous component [15, 16]. Therefore, we developed a hydrogen sulfide detector tube and evaluated its clinical utility. The results demonstrated that the hydrogen sulfide detector tube was effective for objectively identifying “clearly noticeable oral malodor,” defined as malodor exceeding socially acceptable limits.

In developing the detector tube, the detection material was calibrated using standard 200 ppb hydrogen sulfide gas, which corresponds to the human olfactory threshold [12]. However, during clinical evaluation, the sensitivity was reduced, and the detector tube exhibited excellent sensitivity at hydrogen sulfide levels ≧300 ppb, the threshold for clearly noticeable oral malodor [17]. The reduced sensitivity was likely due to interference from moisture and other gases in mouth air [18]. Nevertheless, for general dentists who are not specialists in oral malodor, the ability to objectively identify “clearly noticeable oral malodor” provides a practical basis for referring patients to oral malodor specialists, rather than requiring strict determination of its presence or absence.

The specificity of the hydrogen sulfide detector tube was lower for the OLT score than for the VSC concentration measured by the MS Halimeter. This is because several other components in addition to VSCs, such as ammonia, acetone, indole, and skatole, contribute to oral malodor [19]. The OLT evaluates these odors and can assess a variety of gases, making it the gold standard for oral malodor testing, although there are concerns about the accuracy of judgments that rely on human olfaction [20]. In contrast, because the MS Halimeter targets sulfide ions, it showed a high correlation with hydrogen sulfide, the primary oral malodor gas. In this study, the correlation between the OLT score and the MS Halimeter was 80%. Consistent with our results, previous studies showed correlations of 40%–80% between OLT and other detection methods [10, 21, 22]. This implies that, in ~20% of cases, OLT may detect malodor attributable to non‐VSC compounds that are not captured by sulfide‐specific devices. As with other diagnostic instruments, the present hydrogen sulfide detector should be used in combination with OLT to improve the accuracy of halitosis evaluation.

Various devices for measuring oral malodor are used in clinical practice and research [6–11, 23]. Gas chromatographs and portable gas chromatographs can measure VSC concentrations accurately, but they are expensive and difficult to maintain. Portable sulfide monitors are less costly and easier to operate than gas chromatographs, but their sensors require regular maintenance, such as calibration. ATTAIN, which uses an ammonia detector tube, is also easy to manage and inexpensive, but it requires time for urease activity amplification and additional reagents. In comparison, our hydrogen sulfide detector tube detects hydrogen sulfide directly, allowing the test to be completed in 20s. Another advantage is its ease of explanation to patients, as results are determined by a simple color change in the detector material.

When using our device, care must be taken to prevent saliva from entering the detector tube. Patients were instructed to bite the mouthpiece lightly, gently close their lips, and breathe only through their nose to direct breath into the detector tube. Consequently, most patients were able to perform the test without any problems, and only one patient experienced saliva flow into the tube. When interpreting the color change results proved difficult, comparing the reacted material with unreacted material made judgments easier.

## 5. Conclusions

A new hydrogen sulfide detector tube capable of detecting oral malodor was developed. This detector tube test, when combined with an OLT, should be useful for screening oral malodor in general dental practice.

## Conflicts of Interest

The authors declare no potential conflicts of interest.

## Author Contributions

Nao Suzuki and Jun Takatori designed the study, collected and analyzed the data, and wrote the manuscript. Takashi Hanioka analyzed and interpreted the data. Masahiro Yoneda wrote the Introduction and Discussion. All authors approved the final manuscript and take responsibility for its contents.

## Funding

This study was supported in part by JSPS KAKENHI Grant Numbers 22K10330, 25K13357, and 25K13027, and by the Oral Medicine Research Center of Fukuoka Dental College.

## Data Availability

The data supporting the findings of this study are available from the corresponding author, N.S., upon reasonable request.
