# Correction to “Development of a Simple New Method to Detect Oral Malodor Using a Hydrogen Sulfide Detector Tube”

**DOI:** 10.1155/ijod/9768076

**Published:** 2026-04-21

J. Takatori, N. Suzuki, T. Hanioka, and M. Yoneda, “Development of a Simple New Method to Detect Oral Malodor Using a Hydrogen Sulfide Detector Tube,” *International Journal of Dentistry*, 2025, 2255278, https://doi.org/10.1155/ijod/2255278.

In the article, there were errors in the Results, Figure 3, Table 3, and the affiliations.

In Figure 3, the incorrect image was used, leading to the graph not accurately reflecting the data. The correct Figure 3 is below, and has been updated in the article:

**Figure 3:**
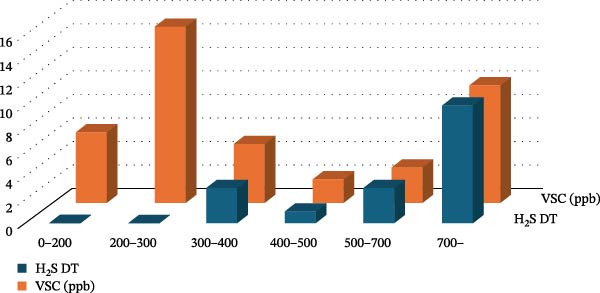
Distribution of VSC concentrations measured using a portable sulfide monitor and corresponding distribution of positive responses with the hydrogen sulfide detector tube. The *X*‐axis shows the VSC level (ppb) measured by the Halimeter (orange bars) and positive H_2_S detection tube (blue bars), and the *Y*‐axis indicates the number of cases.

In Table 3, under the “Judgement criteria” column, “OR” should be replaced with “AND/OR.” The correct Table 3 is shown below, and has been updated in the article:

**Table 3:** Relationship between judgments using the detector tube and the combination of OLT and VSCs (*n* = 37).

Judgment criteria	Sensitivity	False positive rate	Diagnostic performance ^∗^
OLT score ≧ 2.5 AND/OR ≧ 250 ppb	0.64	0.00	0.380
OLT score ≧ 2.75 AND/OR ≧ 250 ppb	0.67	0.00	0.330
OLT score ≧ 3.0 AND/OR ≧ 250 ppb	0.73	0.00	0.270
OLT score ≧ 2.5 AND/OR ≧ 300 ppb	0.80	0.00	0.200
OLT score ≧ 2.75 AND/OR ≧ 300 ppb	0.84	0.00	0.160
OLT score ≧ 3.0 AND/OR ≧ 300 ppb	0.84	0.00	0.160
OLT score ≧ 2.5 AND/OR ≧ 350 ppb	0.79	0.06	0.218
OLT score ≧ 2.75 AND/OR ≧ 350 ppb	0.83	0.05	0.177
OLT score ≧ 3.0 AND/OR ≧ 350 ppb	0.83	0.05	0.177

^∗^Diagnostic performance: lower values indicate better performance.

In the results section:

“The hydrogen sulfide detector tube performed well for OLT scores ≥ 2.75 and MS Halimeter values ≥ 300 ppb.”

should read:

“The hydrogen sulfide detector tube performed well for OLT scores ≥ 2.75 and/or MS Halimeter values ≥ 300 ppb.”

Lastly, the affiliation listed for Masahiro Yoneda was incorrect and has been updated from the “Faculty of Health Care Sciences, Takarazuka University of Medical and Health Care, Takarazuka, Japan” to the ‘Department of General Dentistry, Fukuoka Dental College, Fukuoka, Japan.”

We apologize for these errors.

